# Guillain-Barré Syndrome During the Postpartum Period

**DOI:** 10.7759/cureus.12021

**Published:** 2020-12-10

**Authors:** Mohammed Aabdi, Yassine Mellagui, Amine Bensaid, Houssam Bkiyar, Brahim Housni

**Affiliations:** 1 Anesthesiology, Mohammed VI University Hospital Center, Oujda, MAR

**Keywords:** guillain-barré syndrome, postpartum, case report

## Abstract

Guillain-Barré syndrome (GBS) is an acute peripheral neuropathy that manifests with ascending symmetrical and progressive weakness or paralysis with the absence of reflexes. The incidence of this syndrome in pregnancy and postpartum periods is similar to the general population; however, few published clinical cases have been described. Here, we report the case of a 26-year-old patient who developed GBS with respiratory distress two weeks after her baby's delivery that required long-term mechanical ventilation. The patient's clinical conditions were significantly improved after the use of immunoglobulins and rehabilitation, and she was discharged. Physicians should be aware of the possibility of developing GBS during pregnancy and postpartum periods.

## Introduction

GBS is the first etiology of acute paralytic neuropathy [1]. It can be a life-threatening disease, and it usually appears one to two weeks after an infection or immune stimulation that activates an autoimmune response against peripheral nerves [2,3]. Moreover, it occurs commonly in the third trimester of pregnancy and the first two weeks of the postpartum period [4]. In this case report, we describe a rare clinical case of GBS during the postpartum period that occurred two weeks after her baby's delivery. The patient management required mechanical ventilation for two weeks with excellent improvements after intravenous use of immunoglobulins.

## Case presentation

**Figure 1:**
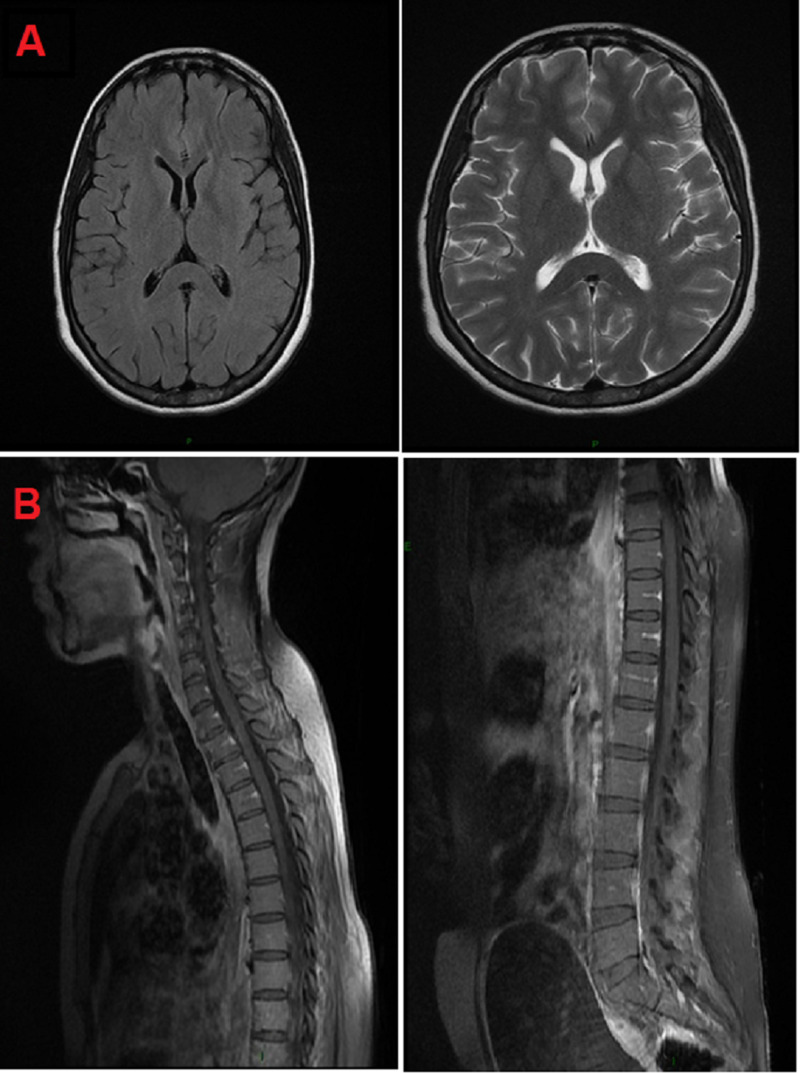
Encephalic (A) and medullary (B) MRI of our patient showing no abnormalities

A 26-year-old woman, primiparous and with no medical history, presented to the emergency department with paresthesia of lower limbs 14 days after her baby's delivery without neuraxial anesthesia. The initial clinical examination found a conscious patient, with power of upper limb of 4/5 and lower limb of 2/5, with no signs of neurological localization or sphincter dysfunction. She was hemodynamically and respiratorily stable (blood pressure of 140/80 mmHg, heart rate at 85 beats/min, and pulse oximetry of 98% at ambient air). Three days later, she developed signs of hypercapnia, including tachycardia, high blood pressure, and alteration of consciousness with dysphagia. At this moment, the patient was intubated and ventilated. The complete blood count was normal (hemoglobin at 12 g/dl, white blood cells at 5200/µL, and sodium at 138 mEq/L). The cerebrospinal fluid analysis showed albuminocytologic dissociation with 5.29 g of proteins and white blood cells of 0/µL. An encephalic and medullary magnetic resonance imaging (MRI) was performed to exclude differential diagnoses, and no pathology was detected (Figure 1).

The patient was treated with five cycles of immunoglobulin (0.5 mg/kg/day) and showed significant improvements. The patient was extubated two weeks after and transferred to the neurology department for rehabilitation.

## Discussion

GBS is a group of autoimmune disease variants with polyradiculoneuropathy [5]. It is usually preceded by infectious diseases or immune stimulations that initiate the autoimmune reactions targeting the peripheral nerves [5]. The immune response is principally associated with humoral immunity mediated by T lymphocytes [6]. The clinical presentation of GBS starts with bilateral symmetrical paresthesia, numbness, and limb weakness, followed by a peak deficit after two to four weeks [2,7,8]. Moreover, the disease progression may vary over six weeks and may include respiratory distress, cardiac arrhythmia, and blood pressure fluctuations [9,10]. The study of cerebrospinal fluid can be normal in the first week but shows albuminocytologic dissociation after two weeks [10,11]. Brain and spinal MRI are indicated to eliminate other causes of polyneuropathy such as subacute compressive myelopathy, transverse myelopathy, and it can show enhancement of spinal roots or cranial nerves in patients with GBS [12,13]. In addition, nerve conduction assessment may be normal in the early stages of the disease, and it shows decreased motor and sensory amplitudes later, with reduced nerve velocity, prolonged F wave latency, increased temporal dispersion, and conduction blocks [14,15]. Medical care and immunological treatments are critical to the management of GBS. The current guidelines recommend respiratory, cardiac, and hemodynamic monitoring to prevent secondary complications [2]. Intravenous immunoglobulins and plasma exchange are widely used and should be started as soon as possible before irreversible nerve damage occurs. Typically, immunoglobulins can be given at a dose of 0.4 g/kg per day for five days or five episodes of plasma exchange over two weeks [2,9,16].

GBS may occur at any time of pregnancy with an increased incidence during the postpartum period and accounts for 1.2-1.9 cases per 100,000 [5,17]. Unfortunately, the diagnosis of GBS is usually delayed in pregnancy and the postpartum period because of the non-specific symptoms [5]. The diagnosis and management of GBS in pregnancy and postpartum is similar to that in the general population [5]. In some cases, the management of this rare presentation requires a transfer to intensive care units for artificial ventilation [18]. To the best of our knowledge, only one case of GBS in a postpartum patient after cesarean section under spinal anesthesia has been described; however, there is no strong evidence supporting the association of neuraxial anesthesia and GBS [19]. Other rare presentations are described in the medical literature, such as relapsing GBS during pregnancy and postpartum and also congenital GBS in a newborn of an affected mother [20].

## Conclusions

GBS, a life-threatening situation, remains underestimated because of its low incidence during the pregnancy and postpartum periods. Physicians should be aware of the possibility of GBS during these critical periods, and GBS should be considered in the differential diagnosis of women who present with neurological symptoms, including paresthesia. Early diagnosis and immune-modulatory therapy are still the backbone of GBS management during pregnancy and postpartum, which improve clinical outcomes for both mother and fetus.
